# Genome-wide investigation of the PLD gene family in alfalfa (*Medicago sativa* L.): identification, analysis and expression

**DOI:** 10.1186/s12864-022-08424-9

**Published:** 2022-03-28

**Authors:** Yuying Yuan, Jinqiu Yu, Lingzelai Kong, Wenkai Zhang, Xiangyin Hou, Guowen Cui

**Affiliations:** Department of Grassland Science, College of Animal Science and Technology, Northeast Agricultural University, Harbin, 150030 China

**Keywords:** MsPLD gene family, Alfalfa, Expression patterns, Abiotic stress, Hormone treatment

## Abstract

**Background:**

External environmental factors, such as salt, alkali and drought, severely limit the acreage and yield of alfalfa. The mining of tolerance-related genes in alfalfa and improving the stress resistance of this plant are essential for increasing alfalfa yield. *PLD* is the main phospholipid hydrolase in plants and plays an important role in plant growth, development, signaling, and resistance to adverse stress. With the availability of whole genome sequences, the annotation and expression of *PLDs* in alfalfa can now be achieved. At present, few studies have investigated *PLDs* in alfalfa. Here, we conducted a study of *PLDs* in alfalfa and identified and analyzed the expression pattern of *PLDs* under different treatments.

**Results:**

Fifty-nine *MsPLDs* were identified in alfalfa and classified into six subtypes: MsPLDα, β, γ, δ and ε belong to the C2-PLD subfamily, and MsPLDζ belongs to the PXPH-PLD subfamily. Members of the same PLD subtype have similar physicochemical properties, sequence structure and domains, but their *cis*-acting elements are different. A qRT-PCR analysis revealed that *MsPLDs* are expressed in multiple tissues. *MsPLDs* can respond to alkali, drought, ABA, IAA, and GA3 treatments and particularly to salt stress. Different expression patterns were found for the same gene under different treatments and different genes under the same treatment. Expression of *MsPLD05* improved salt tolerance in yeast.

**Conclusion:**

This study represents the first genome-wide characterization of *MsPLDs* in alfalfa. Most *MsPLDs* are expressed mainly in mature leaves and respond positively to abiotic stresses and hormonal treatments. This study further expands the resistance gene pool in legume forage grasses and provides a reference for further in-depth study of *MsPLDs* in alfalfa.

**Supplementary Information:**

The online version contains supplementary material available at 10.1186/s12864-022-08424-9.

## Introduction

Phospholipids are not only an important component of cell membranes but also an important source of intracellular signal generation [1]. Phospholipids can be hydrolyzed by phospholipases to produce phosphatidic acid (PA), choline, ethanol and diacylglycerol (DAG). Phospholipases are classified as phospholipase Al (PLA1), phospholipase A2 (PLA2), phospholipase B (PLB), phospholipase C (PLC) and phospholipase D (PLD) depending on the hydrolysis site of glycerophospholipids [1, 2]. Different types of phospholipases show differences in terms of reaction conditions, cofactors and substrate selection [1, 2]. Phospholipase D is the most important phospholipase in plants, can specifically catalyze the hydrolysis of phosphodiester bonds at the end of phospholipid molecules and participates in processes such as plant growth and development [3, 4].

Currently, *PLDs* have been identified in rice (*Oryza sativa*) [5], Arabidopsis (*Arabidopsis thaliana*) [6], soybean (*Glycine max*) [7], grape (*Vitis vinifera*), poplar (*Populus* L*.*) [8] and Chinese plum (*Prunus mume*) [9] plants, which have more than 10 *PLDs*. In Arabidopsis, the PLD gene family is divided into six isoforms based on information on their physicochemical properties and sequence structures: PLDα, β, γ, δ, ε and ζ. However, the N-terminal structural domains of *PLDs* show differences: PLDα, β, γ, δ, and ε contain a C2 structural domain at the N-terminal end and comprise the C2-PLD subfamily, whereas PLDζ has a PX/PH structural domain at the N-terminal end and forms the PX/PH-PLD subfamily. The C2 structural domain is involved in Ca^2+^-dependent and phospholipid binding and all members except PLDα2, β2, γ3 and PLDζ require Ca^2+^ activation, but the amount of Ca^2+^ needed varies among genes [6, 10, 11]. In addition, the PX structural domain is essential for the activity of *PLDs* in the PLDζ subfamily [12, 13].

Several studies have shown that *PLDs* are involved in the production of PAs that are important in plant growth, development, signaling, resistance to adverse stress. Studies on the function of *AtPLDs* have revealed that the functions exercised by different *PLD* isoforms may vary [14, 15]. AtPLDα and δ members are involved in responses to abiotic stresses such as salt and drought [16–22]. In addition, *AtPLDα1* and *AtPLDδ* are involved in stomatal closure, cell senescence, and cell death [21]. *AtPLDε* promotes primary root and root hair elongation under low nitrogen conditions [23, 24]. *AtPLDβ* is associated with the defense response to fungal pathogen infestation [25]. This finding further suggests that different *PLDs* perform unique and important functions in specific plant growth, development or stress response processes. A summary of the mechanism of action of *PLDs* reveals that *PLDs* mainly function by altering the membrane lipid composition, degrading the membrane mass, disrupting membrane function, and participating in cellular regulation as signaling molecules [26]. The most in-depth studies on *PLDα1* in Arabidopsis mainly focused on PLD-protein interactions, including direct protein interactions and protein interactions through PA and other products. At present, some PLD target proteins have been identified, and these include the heterotrimeric G protein Gα subunit (Gα) [27] and the aspartate protease cardosin A [28]. The identified PA target proteins include NADPH oxidase [29], phosphatidylinositol-dependent protein kinase 1 (PDK1) [30], mitogen-activated protein kinase 6 (MPK6) [31], and sphingosine kinase (SPHK) [32]. The abovementioned studies reveal that the PLD- and PA-related signaling pathways form an important component of the plant phospholipid signaling pathway.

Alfalfa is a perennial legume herb with good palatability, high nutritional value and high yield and is known as the “queen of forage grasses” [33]. This herb is also used as an ecological grass to prevent soil erosion and improve soil quality due to its well-developed root system and nitrogen fixation ability [34]. Therefore, alfalfa is widely planted in many countries. Although the PLD gene family has been identified in many plants, no comprehensive study of *PLDs* in alfalfa has been reported thus far. With the release of the alfalfa genome [35], we are better able to systematically investigate the putative functions of *PLDs* in alfalfa. As a result, we successfully identified 59 *MsPLDs* in alfalfa and analyzed the basic physicochemical properties, evolutionary tree, sequence structure, structural domains, and covariance of these 59 *MsPLDs*. These *MsPLDs* were also analyzed in terms of *cis*-acting elements and by qRT-PCR to further clarify their possible functions. The results will lay the foundation for further study of these 59 *MsPLDs* and the mining of resistance gene resources.

## Result

### Identification and characterization of *MsPLDs* in alfalfa

To identify and obtain the *MsPLDs* in the alfalfa genome, a global search of the alfalfa genome using the hidden Markov model (HMM) profile of the HKD domain (PF000164) sequence alignment, conserved structural domain analysis and other methods identified a total of 59 *MsPLDs,* and these *MsPLDs* were used for subsequent analyses (Table 1). Their protein sequences and coding sequences are listed in Additional file 1. *MsPLD01-59* were renamed according to their position on the chromosome, and the names and IDs of the genes are presented in Table 1.

**Table 1:** List of basic information on the 59 *MsPLDs* identified in this study

Gene name	Sequence ID	chromosome	Coordinate (5′-3′)	Protein			Subcellular localization
				**Length(aa)**	**pI**	**MW(kDa)**	
*MsPLD01*	MS.gene005281.t1	chr1.1	61,898,320:61,912,172	1125	5.92	128.59	Cytoplasm; Vacuole
*MsPLD02*	MS.gene40819.t1	chr1.2	59,800,826:59,811,385	1117	5.9	127.60	Cytoplasm
*MsPLD03*	MS.gene23949.t1	chr2.1	1,902,252:1,906,851	667	5.7	75.88	Endoplasmic reticulum; Vacuole
*MsPLD04*	MS.gene00253.t1	chr2.1	57,314,352:57,332,263	1120	6.3	126.94	Cytoplasm
*MsPLD05*	MS.gene36409.t1	chr2.2	2,872,450:2,875,877	756	6.17	86.76	Cytoplasm
*MsPLD06*	MS.gene071874.t1	chr2.2	53,276,118:53,293,622	1119	6.34	126.87	Cytoplasm
*MsPLD07*	MS.gene86393.t1	chr2.3	1,344,450:1,349,273	809	5.5	92.04	Endoplasmic reticulum; Vacuole
*MsPLD08*	MS.gene057038.t1	chr2.3	3,388,802:3,392,217	756	6.17	86.76	Cytoplasm
*MsPLD09*	MS.gene057037.t1	chr2.3	3,400,729:3,404,144	756	6.17	86.76	Cytoplasm
*MsPLD10*	MS.gene50038.t1	chr2.3	3,502,248:3,505,676	756	6.24	86.80	Cytoplasm
*MsPLD11*	MS.gene67327.t1	chr2.3	55,372,147:55,389,928	1119	6.3	126.81	Cytoplasm
*MsPLD12*	MS.gene067395.t1	chr2.4	1,784,846:1,789,476	809	5.5	92.04	Endoplasmic reticulum; Vacuole
*MsPLD13*	MS.gene89026.t1	chr2.4	1,858,953:1,863,743	809	5.5	92.02	Endoplasmic reticulum; Vacuole
*MsPLD14*	MS.gene85200.t1	chr2.4	4,122,792:4,126,221	756	6.17	86.73	Cytoplasm
*MsPLD15*	MS.gene038940.t1	chr2.4	55,568,036:55,587,627	1119	6.26	126.82	Cytoplasm
*MsPLD16*	MS.gene32629.t1	chr3.1	84,952,195:84,955,138	783	6.37	88.92	Endoplasmic reticulum; Vacuole
*MsPLD17*	MS.gene32627.t1	chr3.1	84,968,523:84,971,756	825	5.92	93.83	Endoplasmic reticulum; Vacuole
*MsPLD18*	MS.gene52800.t1	chr3.1	90,894,759:90,901,718	851	8.31	96.65	Cytoplasm
*MsPLD19*	MS.gene072896.t1	chr3.2	87,203,145:87,206,378	825	5.92	93.83	Endoplasmic reticulum; Vacuole
*MsPLD20*	MS.gene072894.t1	chr3.2	87,219,454:87,222,732	826	6.46	93.45	Endoplasmic reticulum; Vacuole
*MsPLD21*	MS.gene014379.t1	chr3.2	90,761,398:90,768,367	851	8.19	96.73	Cytoplasm
*MsPLD22*	MS.gene014832.t1	chr3.3	87,825,233:87,828,519	826	6.24	93.47	Endoplasmic reticulum; Vacuole
*MsPLD23*	MS.gene014830.t1	chr3.3	87,840,038:87,843,321	825	6.01	93.84	Endoplasmic reticulum; Vacuole
*MsPLD24*	MS.gene69358.t1	chr3.3	93,688,962:93,695,958	851	8.19	96.70	Cytoplasm
*MsPLD25*	MS.gene67061.t1	chr3.4	93,241,223:93,245,494	796	6.59	90.01	Endoplasmic reticulum; Vacuole
*MsPLD26*	MS.gene67059.t1	chr3.4	93,258,522:93,262,462	825	5.92	93.83	Endoplasmic reticulum; Vacuole
*MsPLD27*	MS.gene064799.t1	chr3.4	98,282,304:98,289,298	851	8.19	96.70	Cytoplasm
*MsPLD28*	MS.gene026763.t1	chr4.1	85,441,869:85,445,416	808	5.58	91.68	Endoplasmic reticulum; Vacuole
*MsPLD29*	MS.gene47888.t1	chr4.1	11,906,321:11,909,993	828	7.22	93.51	Cytoplasm
*MsPLD30*	MS.gene75365.t1	chr4.2	89,727,307:89,730,857	808	5.54	91.58	Endoplasmic reticulum; Vacuole
*MsPLD31*	MS.gene030945.t1	chr4.3	11,556,291:11,559,963	828	7.22	93.51	Cytoplasm
*MsPLD32*	MS.gene052137.t1	chr4.3	11,572,534:11,576,203	828	7.22	93.51	Cytoplasm
*MsPLD33*	MS.gene052138.t1	chr4.3	11,585,594:11,589,266	828	7.22	93.51	Cytoplasm
*MsPLD34*	MS.gene052139.t1	chr4.3	11,597,424:11,600,973	787	8.19	89.17	Cytoplasm
*MsPLD35*	MS.gene030946.t1	chr4.3	11,612,495:11,616,167	828	7.22	93.51	Cytoplasm
*MsPLD36*	MS.gene60041.t1	chr4.3	86,121,962:86,125,511	808	5.58	91.68	Endoplasmic reticulum; Vacuole
*MsPLD37*	MS.gene27931.t1	chr4.4	12,348,219:12,351,956	828	7.07	93.57	Cytoplasm
*MsPLD38*	MS.gene034074.t1	chr4.4	88,855,262:88,858,802	808	5.62	91.68	Endoplasmic reticulum; Vacuole
*MsPLD39*	MS.gene64823.t1	chr5.1	13,898,847:13,907,069	869	6.67	98.53	Cytoplasm
*MsPLD40*	MS.gene20350.t1	chr5.1	4,652,023:4,656,053	800	7.85	90.53	Cytoplasm
*MsPLD41*	MS.gene20351.t1	chr5.1	4,658,972:4,663,281	848	7.02	95.59	Cytoplasm
*MsPLD42*	MS.gene22597.t1	chr5.2	3,876,181:3,880,214	800	7.35	90.48	Cytoplasm
*MsPLD43*	MS.gene22596.t1	chr5.2	3,882,132:3,887,090	802	6.88	90.62	Cytoplasm
*MsPLD44*	MS.gene67570.t1	chr5.2	14,035,406:14,043,438	869	6.59	98.54	Cytoplasm
*MsPLD45*	MS.gene67512.t1	chr5.2	14,068,746:14,076,357	607	6.24	68.53	Cytoplasm
*MsPLD46*	MS.gene025734.t1	chr5.3	4,671,633:4,675,827	854	7.18	96.32	Cytoplasm
*MsPLD47*	MS.gene025735.t1	chr5.3	4,677,864:4,682,196	848	6.69	95.58	Cytoplasm
*MsPLD48*	MS.gene063676.t1	chr5.3	13,420,601:13,428,164	869	6.67	98.52	Cytoplasm
*MsPLD49*	MS.gene015516.t1	chr5.4	5,638,597:5,642,787	862	7.62	97.22	Cytoplasm
*MsPLD50*	MS.gene015515.t1	chr5.4	5,644,824:5,649,159	848	6.77	95.57	Cytoplasm
*MsPLD51*	MS.gene42387.t1	chr7.1	27,281,427:27,286,953	1112	7.57	124.38	Cytoplasm
*MsPLD52*	MS.gene27622.t1	chr7.2	29,824,181:29,829,710	1113	7.57	124.47	Chloroplast; Cytoplasm
*MsPLD53*	MS.gene42212.t1	chr7.3	29,807,554:29,813,088	1113	7.57	124.44	Chloroplast; Cytoplasm
*MsPLD54*	MS.gene96040.t1	chr7.4	29,490,914:29,496,124	995	7.96	111.18	Cytoplasm
*MsPLD55*	MS.gene019429.t1	chr8.1	68,377,847:68,384,748	1045	6.67	117.16	Chloroplast; Cytoplasm
*MsPLD56*	MS.gene072112.t1	chr8.1	81,395,694:81,405,783	921	5.8	106.32	Cytoplasm; Vacuole
*MsPLD57*	MS.gene36927.t1	chr8.2	64,475,923:64,482,575	1047	6.67	117.35	Chloroplast; Cytoplasm
*MsPLD58*	MS.gene37538.t1	chr8.3	60,823,389:60,830,276	1049	6.68	117.65	Chloroplast; Cytoplasm
*MsPLD59*	MS.gene63691.t1	chr8.4	62,837,893:62,844,731	1028	6.72	115.28	Chloroplast; Cytoplasm

The main information regarding the gene locations, subcellular localizations and physicochemical properties of the proteins are also presented in Table 1. The *MsPLDs* were unevenly distributed on all chromosomes except chromosome 6. The analysis of the distribution of the genes revealed that chromosomes 1, 7 and 8 had a sparser distribution, with 2, 4 and 4 *MsPLDs*, respectively, whereas each of the remaining chromosomes had more than 10 *MsPLDs*. Subcellular localization predictions showed that most of the *MsPLDs* were distributed in the cytoplasm, followed by the vacuole, endoplasmic reticulum and chloroplast. Therefore, we hypothesized that *MsPLDs* mainly exercise functions in the cytoplasm. The analysis of the physicochemical properties of the proteins relative differences in terms of their sequence length, isoelectric point (pI) and molecular weight within the ranges of 607 to 1125 aa, 5.5 to 8.31 and 68.53 to 128.59 kDa, respectively. More than half of the sequences had a length of 800–900 aa. With the exception of *MsPLD18*, *MsPLD21*, *MsPLD24*, *MsPLD27*, and *MsPLD34*, the pI of all the genes was less than 8.0.

### Phylogenetic and protein sequence analysis of the *MsPLD* gene family

**Fig. 1:**
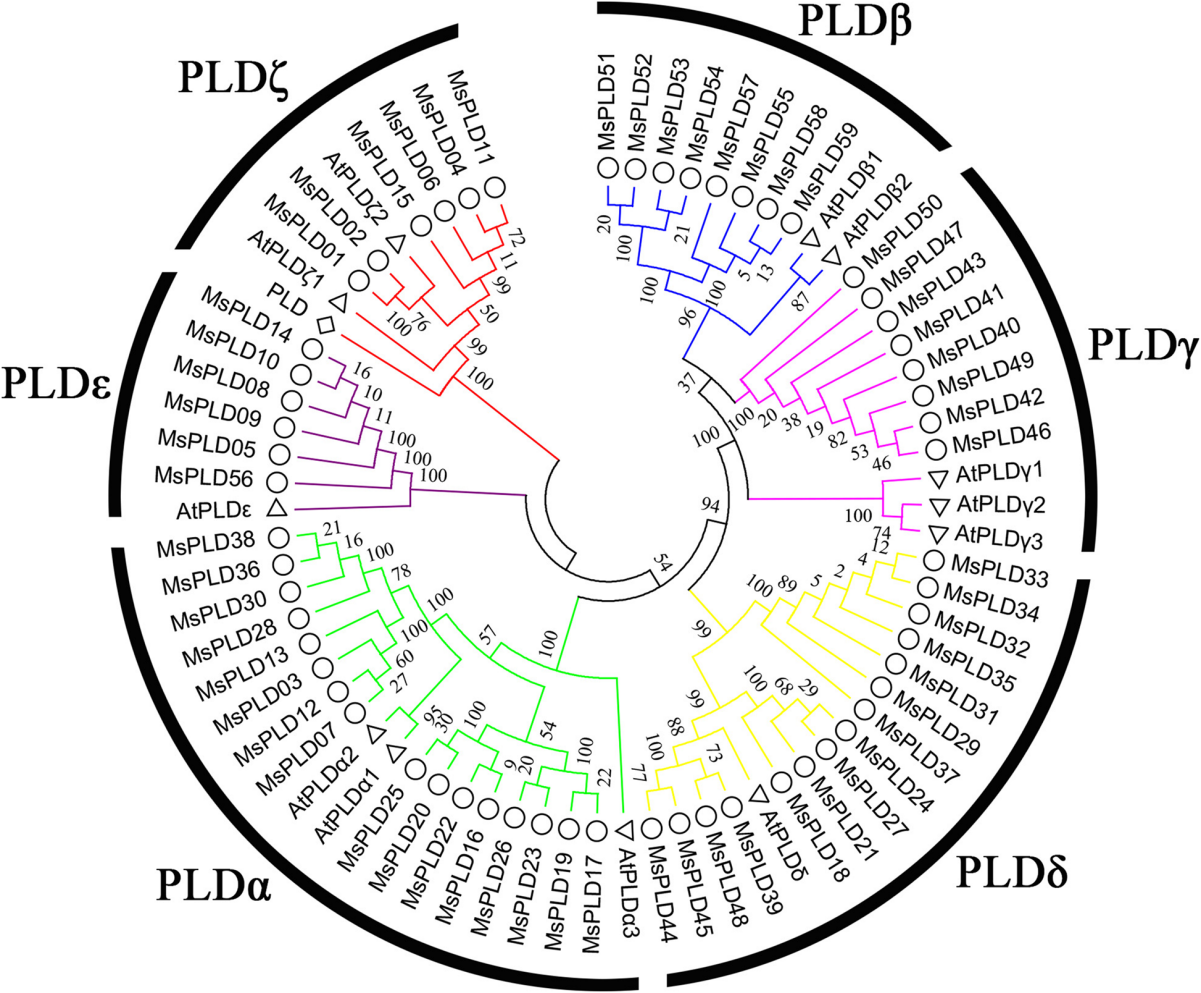
Phylogenetic analysis of *PLDs* in alfalfa and Arabidopsis. The amino acid sequences of PLD proteins from alfalfa and Arabidopsis were used for the phylogenetic analysis. The phylogenetic tree was constructed with MEGA 6.0 using the maximum likelihood method with 1000 bootstrap replicates. The different colors on the evolutionary tree branches represent different PLD subfamilies, and the symbols ○, △ and ◇ represent PLDs in alfalfa, Arabidopsis, and Drosophila, respectively

To investigate the evolutionary relationships of MsPLD gene family members, a phylogenetic tree was constructed using amino acid sequences of Arabidopsis and alfalfa (Fig. 1). The results showed that the 59 *MsPLDs* in alfalfa were divided into six isoforms, namely MsPLDα, β, γ, δ, ε, and ζ. This result was consistent with findings in Arabidopsis, where the maximum number of MsPLDα was 16, and the minimum number of MsPLDζ and MsPLDε were 6 in alfalfa.

**Fig. 2:**
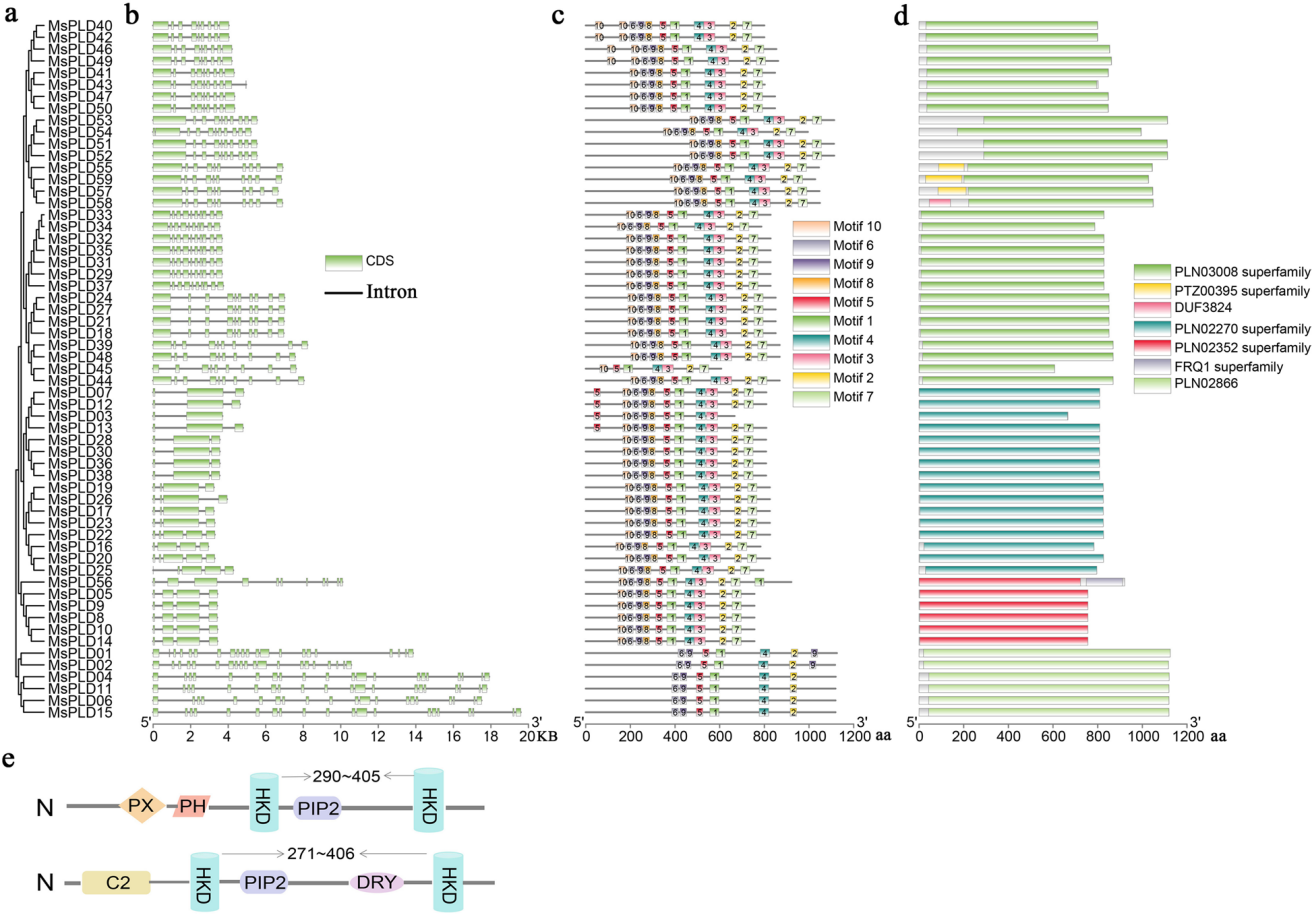
Sequence structure analysis of *MsPLDs* in alfalfa. **a** Phylogenetic analysis of the amino acid sequences of 59 *MsPLDs* using MEGA 6.0. **b** Exon–intron structure of *MsPLDs*. **c** Motif distribution of MsPLD proteins. Different motifs (1–10) are indicated by different colors. The sequence logos and information for each motif are provided in Additional files 2 and 3. **d** Domain distribution of MsPLD proteins. Different domains are indicated by different colors. **e** Schematic depiction of alfalfa MsPLD domain structures

The diversity of the gene structures supported phylogenetic grouping to some extent [36]. To better show the sequence structure of *MsPLDs* in alfalfa, we constructed a phylogenetic evolutionary tree using the protein sequences of 59 *MsPLDs* (Fig. 2a). The comparison of the exon–intron organizations of different *MsPLDs* showed that the gene structures of *MsPLDs* were relatively different but similar among the same isoforms (Fig. 2b). Among the *MsPLDs*, gene members belonging to the MsPLDβ, γ and δ subtypes all contain 8 or 9 introns. In contrast, the number of introns in the MsPLDα members ranged from 1 to 4. With the exception of *MsPLD56*, all other members of the MsPLDε subtype contained 3 introns. The maximum number of introns obtained for the MsPLDζ subtype was between 18 and 20. We speculate that this finding is related to the longer sequences of the MsPLDζ subtype members.

To further investigate the structural features of alfalfa PLD proteins, the conserved motifs were analyzed. A total of 10 conserved motifs were obtained (Fig. 2c). The logos and basic information of these base sequences are shown in Additional files 2 and 3. All members of the MsPLDα, β, γ, δ and ε subtypes contained motifs 1–10 with the exception of *MsPLD03,* which did not contain motifs 2 and 7, and *MsPLD48,* which did not harbor motif 8. In contrast, the members of the MsPLDζ subtype contained only motifs 1, 4, 5, 6 and 9. A CDD analysis revealed that the 59 *MsPLDs* contained a total of 7 domains, and all of these proteins were related to phospholipase D (Fig. 2d). The subsequent analysis using the Pfam and InterPro websites revealed that the PLDζ subtype members all contained the PXPH structural domain and classified these members into the PXPH-PLD subfamily, whereas the remaining five subtypes all harbored the C2 structural domain and were classified into the C2-PLD subfamily. Subsequent sequence alignment revealed that the two HKD structural domains were highly conserved (except for the mutation of D in the second HKD structural domain of *MsPLD03* to K and the deletion of D in the second HKD structural domain of *MsPLD56*) and separated by 271–400 amino acids (Additional file 4). The structures predicted for the two PLD subfamilies are shown in Fig. 2e. These results are consistent with the classification results obtained in species such as Arabidopsis. We thus hypothesize that *PLDs* are highly conserved in plants and the location of each structural domain varies from species to species.

### Collinearity and gene duplication analysis of *MsPLDs*

**Fig. 3:**
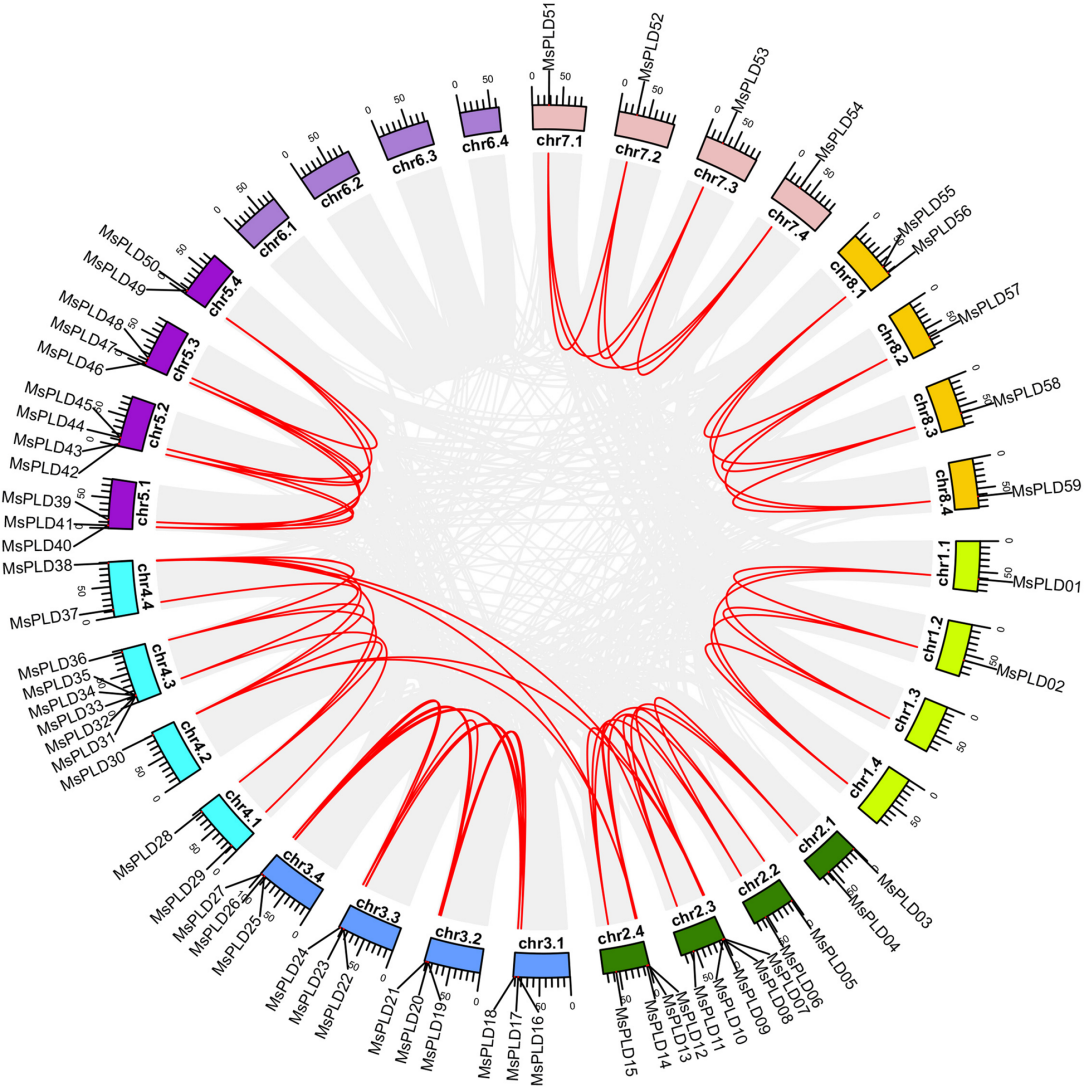
Gene duplication analysis of the *MsPLDs*. The 59 *MsPLDs* were mapped to 7 chromosomes. The gray lines indicate all synteny blocks in the alfalfa genome, and the duplicated gene pairs of *MsPLDs* are connected with red lines

Gene duplication events have played an important role in the expansion of many gene families; therefore, a homologous BLAST of amino acids of alfalfa was performed with the MCScan toolkit, and 139,504 synteny gene pairs and 9733 groups of tandem duplication genes were identified at the genome level in alfalfa. The MsPLD gene family of synteny gene pairs and tandem duplication gene pairs were selected and visualized using TBtools (Fig. 3 and Additional file 5). In the MsPLD gene family of alfalfa, 59 synteny gene pairs including 4 pairs of segmental duplication gene events, were identified. In addition, 8 tandem duplication events occurred. Information on these gene pairs is provided in Additional file 5. Replication events regarding *PLDs* have not been reported in Arabidopsis. Thus, the findings demonstrate that gene duplication events enabled expansion of the MsPLD gene family in alfalfa, which may also explain why the number of *PLDs* in alfalfa is markedly higher than that in Arabidopsis.

The Ka, Ks and Ka/Ks of the *MsPLD* gene pairs were calculated to study the evolutionary functional constraints in alfalfa (Additional file 6). In the present study, the Ka values for each gene pair were found to be in the range of 0 to 0.083 whereas Ks values ranged from 0 to 0.80. All of the MsCSase gene pairs with Ka/Ks > 1 were subjected to positive selection. The results suggest that purifying selection was the main force driving the evolution of *MsPLDs*.

### Regulatory elements in the *MsPLD* promoters

**Fig. 4:**
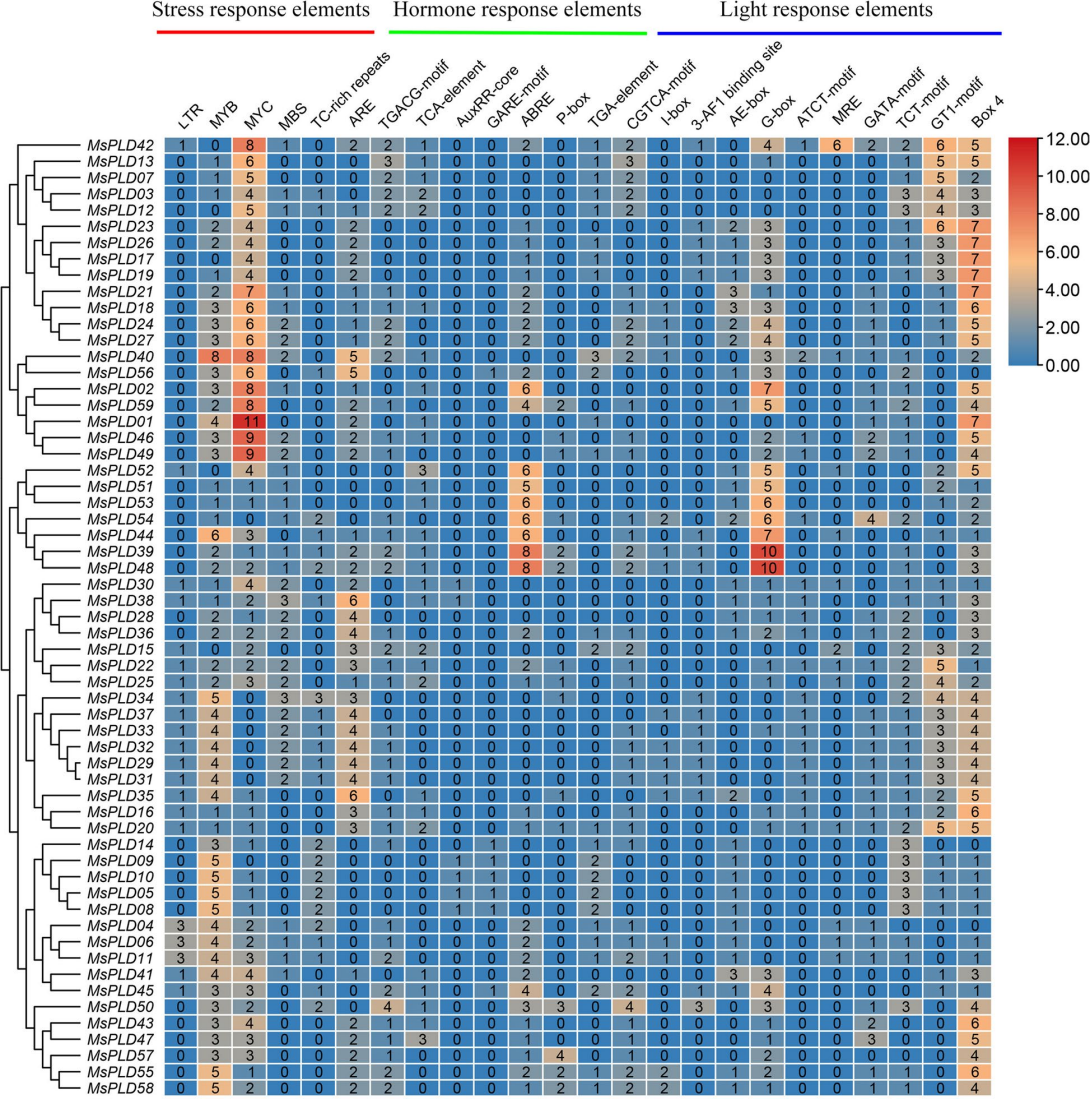
*Cis*-elements in putative promoter regions of *MsPLDs* in alfalfa. The numbers in the boxes indicate the number of *cis*-acting elements

To identify putative *cis*-elements involved in *MsPLD* transcriptional regulation, a 2.0-kb promoter region upstream from the ATG translation start codon of each *MsPLD* was analyzed. We selected 24 major putative *cis*-elements and grouped them into three categories: 6 *cis*-elements that respond to stress, 8 *cis*-elements that respond to hormones, and 10 light-responsive elements (Fig. 4). DRE, MYB and MYC are typical promoter *cis*-elements involved in abiotic stress induction in plants and can positively respond to stresses such as high salt, low temperature and drought. The hormone-responsive elements identified were associated with hormones such as salicylic acid, anxin, gibberelline and abscisic acid. In addition, MYB, MYC, ARE, ABRE, G-box, GT1 motif and Box 4 were the most abundant. However, no clear correlation was found between the type and number of *cis*-acting elements and the distribution of subfamily members. Thus, we speculate that *MsPLDs* may be involved in abiotic stress and hormonal regulation, and different members of the same subfamily may have different response patterns.

### qRT-PCR analysis of *MsPLDs* in different samples

**Fig. 5:**
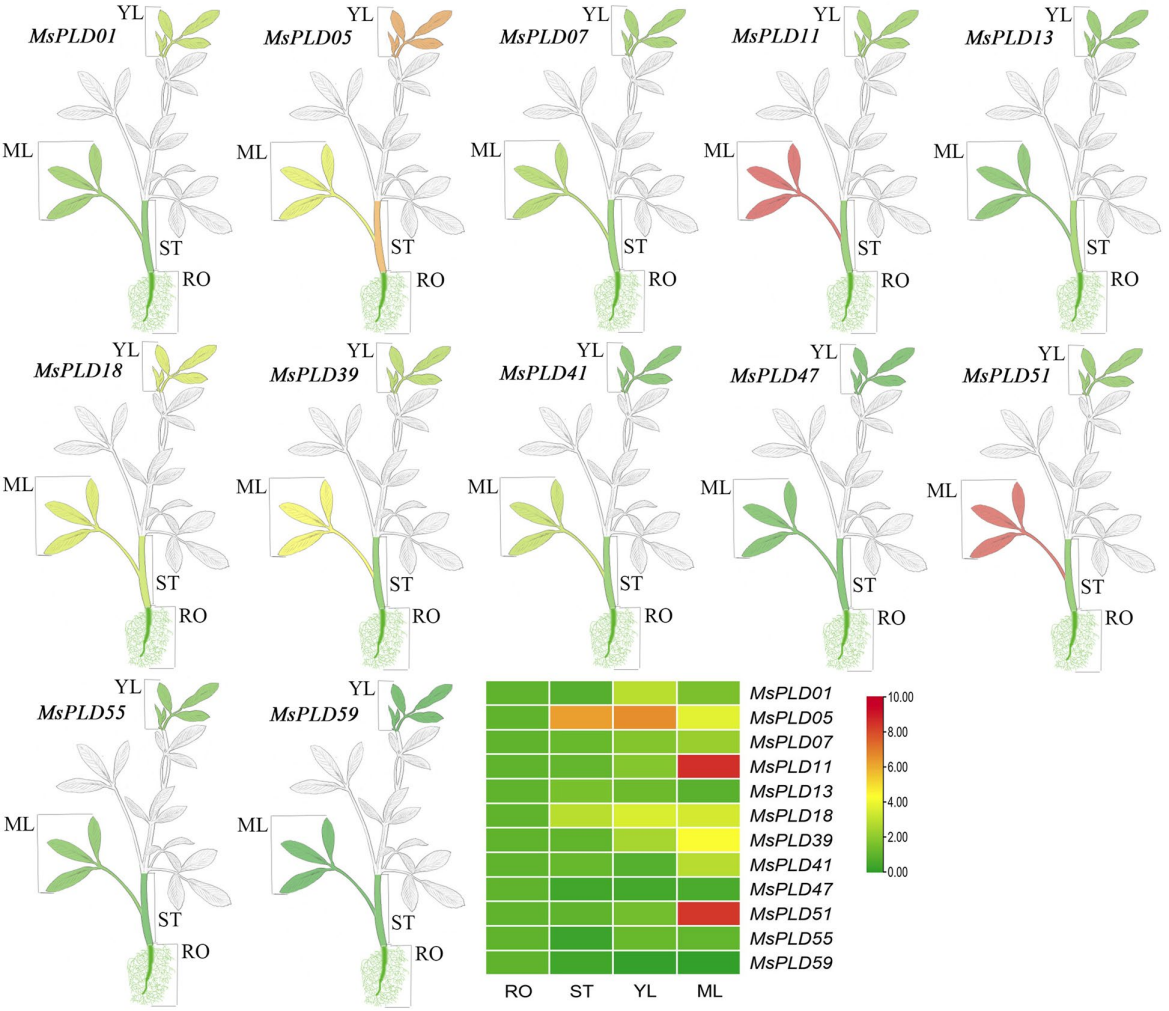
Analysis of the expression patterns in different alfalfa tissues. YL: Young leaf; MF: mature leaf; ST: stem; and RO: root. Different lowercase letters indicate that the difference is significant (*P* < 0.05); and this statement also applies to subsequent figures

To further clarify the potential functions that may exist for *MsPLD*s, we performed a qRT-PCR analysis of different samples of alfalfa. First, twelve *MsPLDs* were selected from different subfamilies based on the phylogenetic analysis and the analysis of the *cis*-acting elements in the promoter region. The experimental results (Fig. 5) showed that the 12 selected *MsPLDs* were expressed in the roots, stems and leaves of alfalfa. These results are consistent with findings in Arabidopsis [23, 37]. *PLDs* can be expressed in multiple plant tissues. *MsPLD47* and *MsPLD59* were mainly expressed in roots, whereas the other 10 *MsPLDs* were mainly expressed in leaves, and the levels of the same gene showed differences between young and mature leaves. *MsPLD05* was similarly expressed at higher levels in stems and young leaves. This finding may be related to the fact that different genes act in different tissues.

**Fig. 6:**
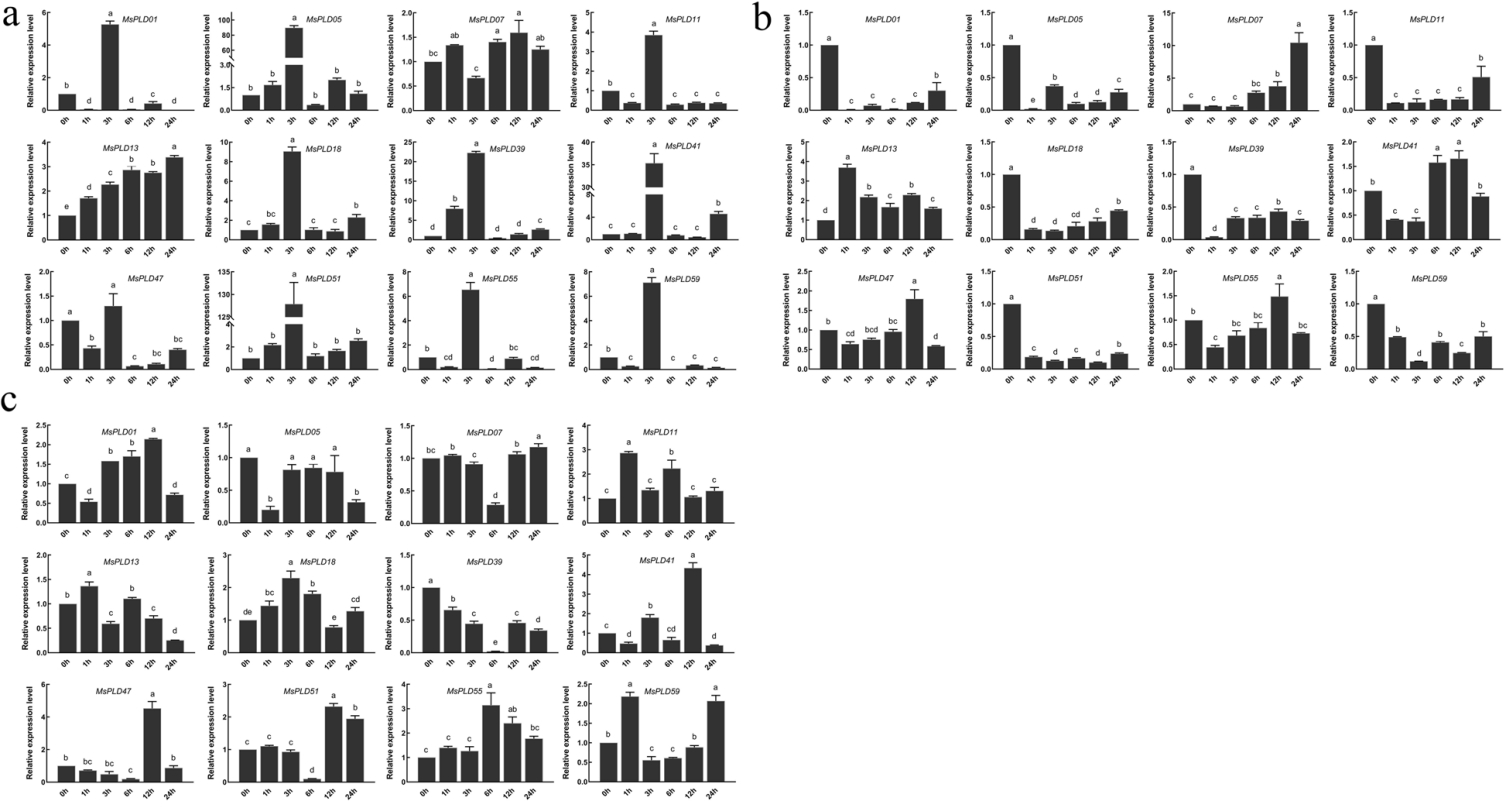
Analysis of expression patterns under abiotic stresses. **a**, **b**, and **c** represent the expression patterns of genes under salt, alkali and drought stresses, respectively

Based on the results from the *cis*-acting element analysis and studies of the PLD gene family in Arabidopsis, we determined the relative expression of 12 *MsPLD* genes under various abiotic stresses and different hormone treatments. A qRT-PCR analysis under salt, alkali, and drought stresses revealed that *MsPLDs* responded to abiotic stresses through different expression patterns (Fig. 6a-c). Most of the *MsPLDs* responded to drought and salt stresses via upregulation (Fig. 6a, c), whereas under alkali stress, most *MsPLDs* showed a downregulated expression pattern (Fig. 6b). Under salt stress, all *MsPLDs* except *MsPLD07* and *MsPLD13* showed the highest expression at 3 h, whereas no obvious change was detected under drought and alkali stresses. A comparison of the expression patterns among different subfamily members under the same stress treatment revealed that most subfamily members presented similar expression patterns, for example, MsPLDα, β, δ and ζ under drought stress, MsPLDα, γ, δ and ζ under alkali stress and MsPLDβ, γ, δ and ζ under salt stress. The remaining subfamily members exhibited similar expression patterns among the differential expression patterns of the remaining subfamily members and need to be further investigated. The expression pattern of *MsPLD05* was most representative of the three stresses: the relative expression of this gene significantly upregulated under salt stress, was not significantly altered under drought stress but was significantly downregulated in response to alkali stress.

**Fig. 7:**
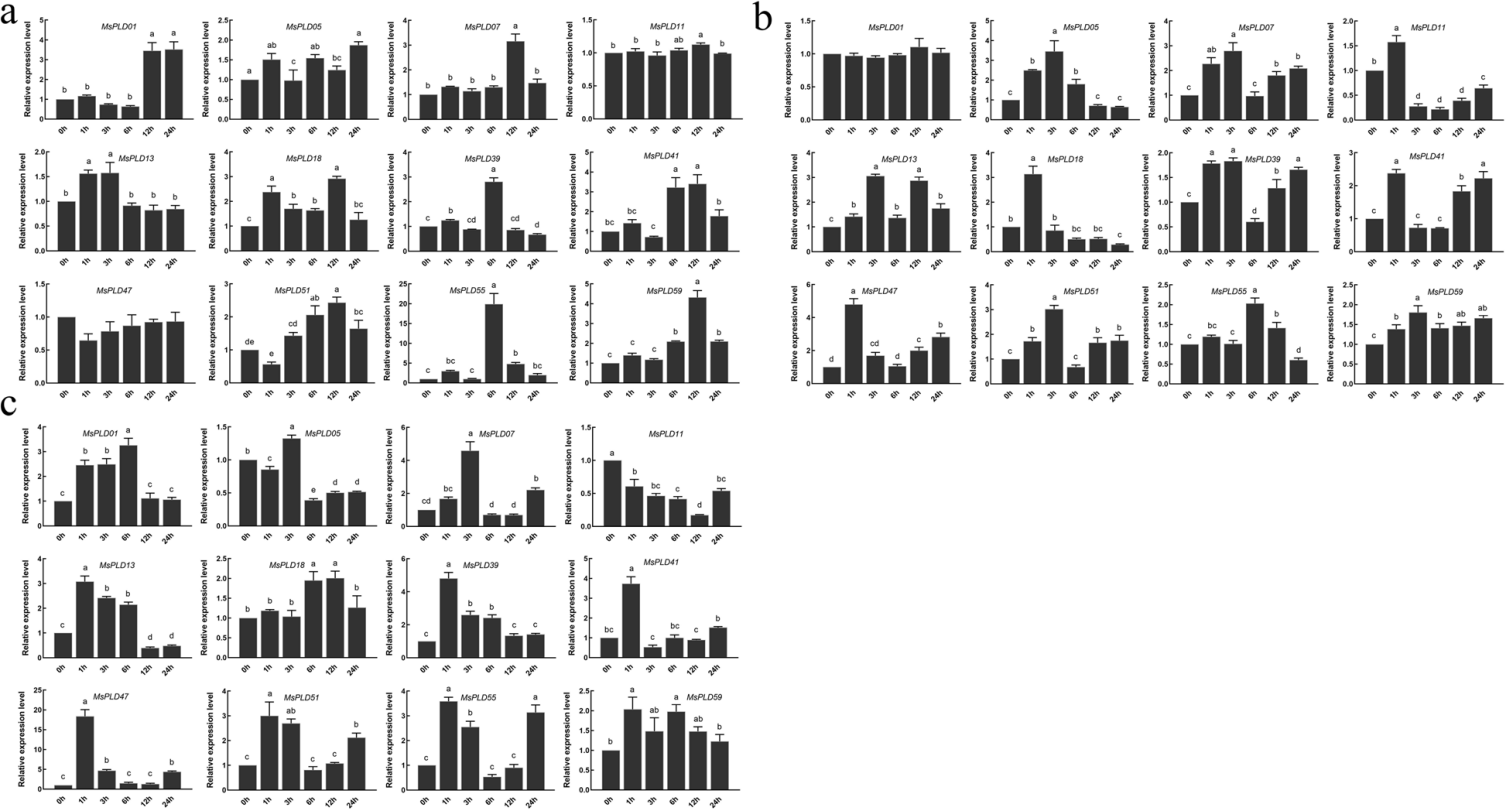
Analysis of expression patterns under hormone treatment. **a**, **b**, and **c** represent the expression patterns of genes under ABA, IAA, and GA3 treatment, respectively

To better understand the role of *MsPLDs* in phytohormone signaling pathways, a qRT-PCR analysis of alfalfa under ABA, IAA and GA3 hormone treatments (Fig. 7a-c) was performed, and the results revealed that the *MsPLD07*, *MsPLD13*, *MsPLD51*, *MsPLD55*, *MsPLD59*, and *MsPLD41* genes could respond positively to these three hormone treatments. With the exception of the downregulated trend observed for *MsPLD11* under GA3 treatment, all the responding genes roughly showed upregulated expression, but the expression levels of same genes under different hormone treatments and those of different genes under the same hormone treatments exhibited differences. For example, the relative expression of *MsPLD01* was significantly upregulated after 12 h and 24 h of ABA treatment, whereas the relative expression of *MsPLD13* was significantly upregulated after 1 and 3 h of this treatment. The relative expression of *MsPLD13* was significantly upregulated after 12 and 24 h of ABA treatment. Although both genes were upregulated in response to stress, some responded during the pretreatment period, whereas others exhibited responses in the posttreatment period. Taken together, the results reveal that *MsPLDs* can respond to hormone treatment and that the responsive genes show differences at different time points.

### Expression of *MsPLD05* improves salt tolerance in yeast

**Fig. 8:**
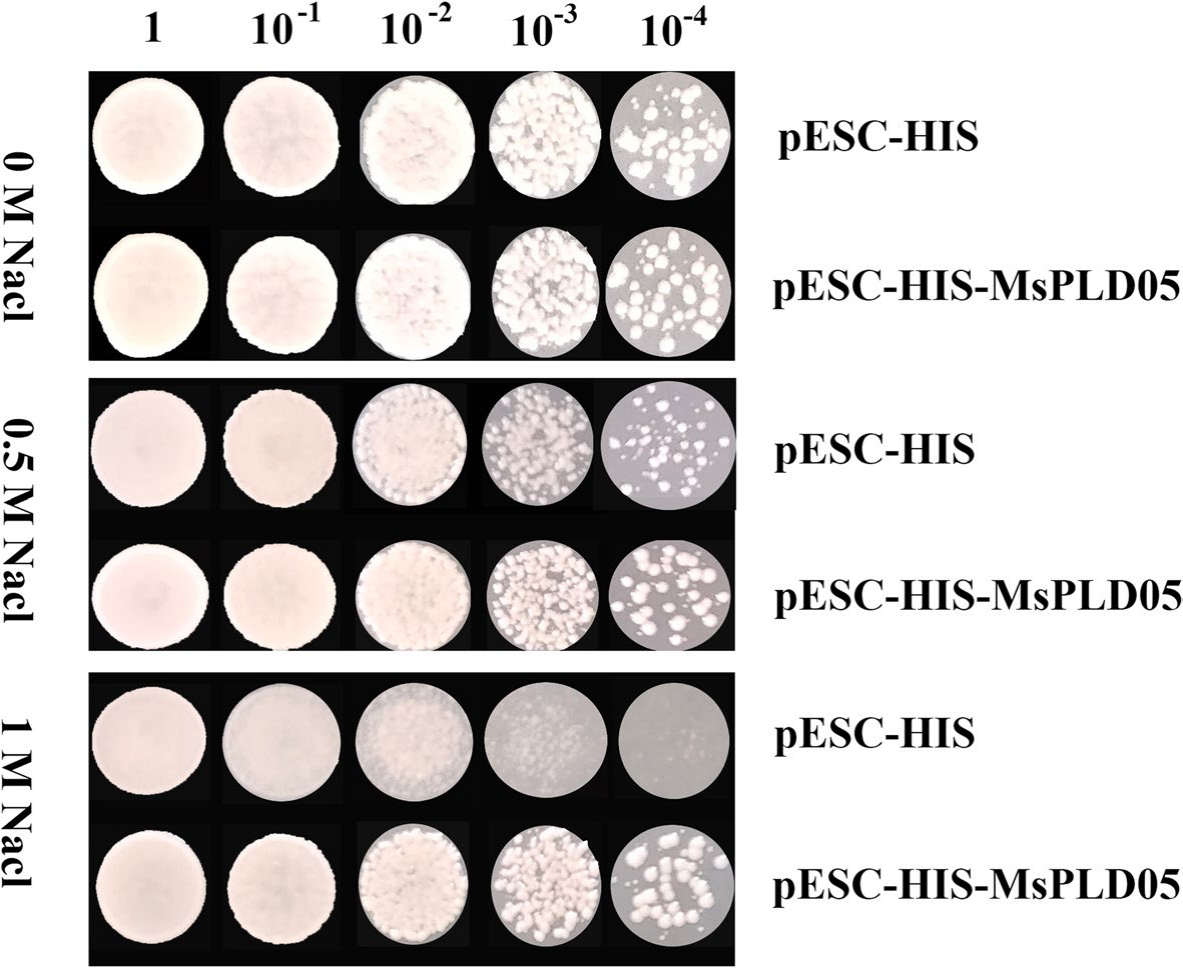
Phenotypic identification of the yeast strain Y187 transformed with *MsPLD05* under salt stress

Combining the results of sequence analysis and qRT-PCR experiments, we finally cloned *MsPLD05* and expressed it in yeast. Under normal growth conditions, there was no significant phenotypic difference between yeast containing pESC-HIS and pESC-HIS-MsPLD05. However, under salt stress, pESC-HIS-MsPLD05 yeast had a better phenotype, and the phenotypic differences became more pronounced with increasing salt concentrations (Fig. 8). This result also implies that this gene can respond positively to this stress and can improve stress resistance when expressed.

## Discussion

Phospholipase D is the most important class of phospholipid hydrolases in plants and plays an important role in regulating cell membrane lipid metabolism, participating in plant growth and development and responding to stress [3, 4, 18, 20, 29, 38]. To date, most studies on *PLDs* have focused on responses to specific stresses, such as salt, cold, and ABA. However, the identification of *PLDs* in alfalfa and their response under multiple stresses have not been reported. Therefore, in this study, the *PLDs* in alfalfa were identified and analyzed with respect to their bioinformatics and expression patterns under various stresses. The present study has furthered our understanding of *MsPLDs* and provides insight into the functions of *MsPLDs*.

### Evolution of the alfalfa *MsPLD* gene family

The number of *PLDs* varies among species. Fewer than 20 *PLDs* have been identified in other species, such as Arabidopsis (*Arabidopsis thaliana*), rice (*Oryza sativa* L.), and apple (*Malus* × *domestica*) [5, 9, 39]. A total of 59 *MsPLDs* were identified in the alfalfa genome. The number of *PLDs* in alfalfa was markedly higher than that in other diploid plants. We speculate that this result was obtained because alfalfa is a tetraploid plant, whereas all ither abovementioned plants are diploid; in addition, our identification results may include alleles, which would, in turn, leads to an excess number of *PLDs* in alfalfa. In addition, a comparison of the gene duplication events of *PLDs* (Fig. 3) revealed more tandem duplication events and synteny duplication events in alfalfa, which also directly leads to an increase in the number of *PLDs* in alfalfa.

The 59 *PLDs* in alfalfa were classified into six isoforms, MsPLDα, β, γ, δ, ε, and ζ, and into two subfamilies according to differences in their structural domains (Fig. 1). Members of the same isoform have similar sequence lengths, physicochemical properties, gene structures and motif distributions; however, differences in these features were observed among different isoforms, and the largest differences were detected between members of the MsPLDζ isoform and the other isoforms (Table 1, Fig. 2b-d). This finding may be related to differences in the N-terminal structural domain. These results also further support the results from the evolutionary analysis. Although the gene classification results were consistent with the results obtained in Arabidopsis, the gene structural domain analysis revealed that the two were more different, with 271–407 amino acids between the two HKD structural domains in alfalfa (Fig. 2e) and approximately 320 amino acids between the two HKD structural domains in Arabidopsis [6, 39]. This finding suggests that although *PLDs* are highly conserved in plants, they show differences among species, but whether these differences lead to new functions of *PLDs* in alfalfa needs to be further investigated. Duplication events in the active regions, the coding sequence (CDS), and/or the regulatory sequence, the promoter region, can cause members of a gene family to acquire new functions [40, 41].

### Potential roles of *MsPLDs* in alfalfa

The tissue-specific expression analysis of *MsPLDs* revealed that the 12 selected genes were expressed in different tissues, but the majority of the *MsPLDs* were highly expressed in leaves, and only *MsPLD47* and *MsPLD59* were mainly expressed in roots (Fig. 5). This finding also implies that *MsPLD47* and *MsPLD59* may exercise their functions mainly in the roots. Interestingly, the subcellular localization prediction shows that *MsPLD59* is localized in chloroplasts and the cytoplasm (Table 1). It has been shown that the subcellular localization of genes is affected by the growth period of plants, and different developmental stages are specifically expressed in different tissues [42]. Whether this finding is also the case for *MsPLD59* and whether *MsPLD59* functions in alfalfa roots need to be further verified. In addition, the relative expression of the same gene showed differences between young and mature leaves, which suggests that *MsPLDs* regulate the growth and development of alfalfa leaves through different expression patterns at different developmental periods. Overall, most of the *MsPLDs* were localized in the leaves, where they exert their actions.

We performed a qRT-PCR analysis of alfalfa under abiotic stress and hormone treatments, and this expression pattern analysis can provide insight into the potential functions of the MsPLD gene family. The experimental results showed that *PLDs* could respond positively to abiotic stress and hormone treatments, and the expression patterns of *PLDs* exhibited differences between different treatments (Figs. 6 and 7). The *MsPLDs* responded most significantly to salt stress among the different treatments, which was similar to the findings obtained in Arabidopsis. The exposure of Arabidopsis to salt stress activates and induces *PLDα1* to produce PA in the plant, and this step is followed by activation of related enzymes downstream of PA [20, 31, 43–45], whereas *AtPLDα3* regulates the salt stress response by promoting root growth [37]. This finding shows that different *PLDs* have different modes of action in response to salt stress. Based on this result, we hypothesized that different *MsPLDs* may have different modes of action under the same stress, which subsequently leads to different expression patterns. Thus, whether the same genes that show different expression patterns when subjected to different stresses also have different modes of action needs to be further investigated.

**Fig. 9:**
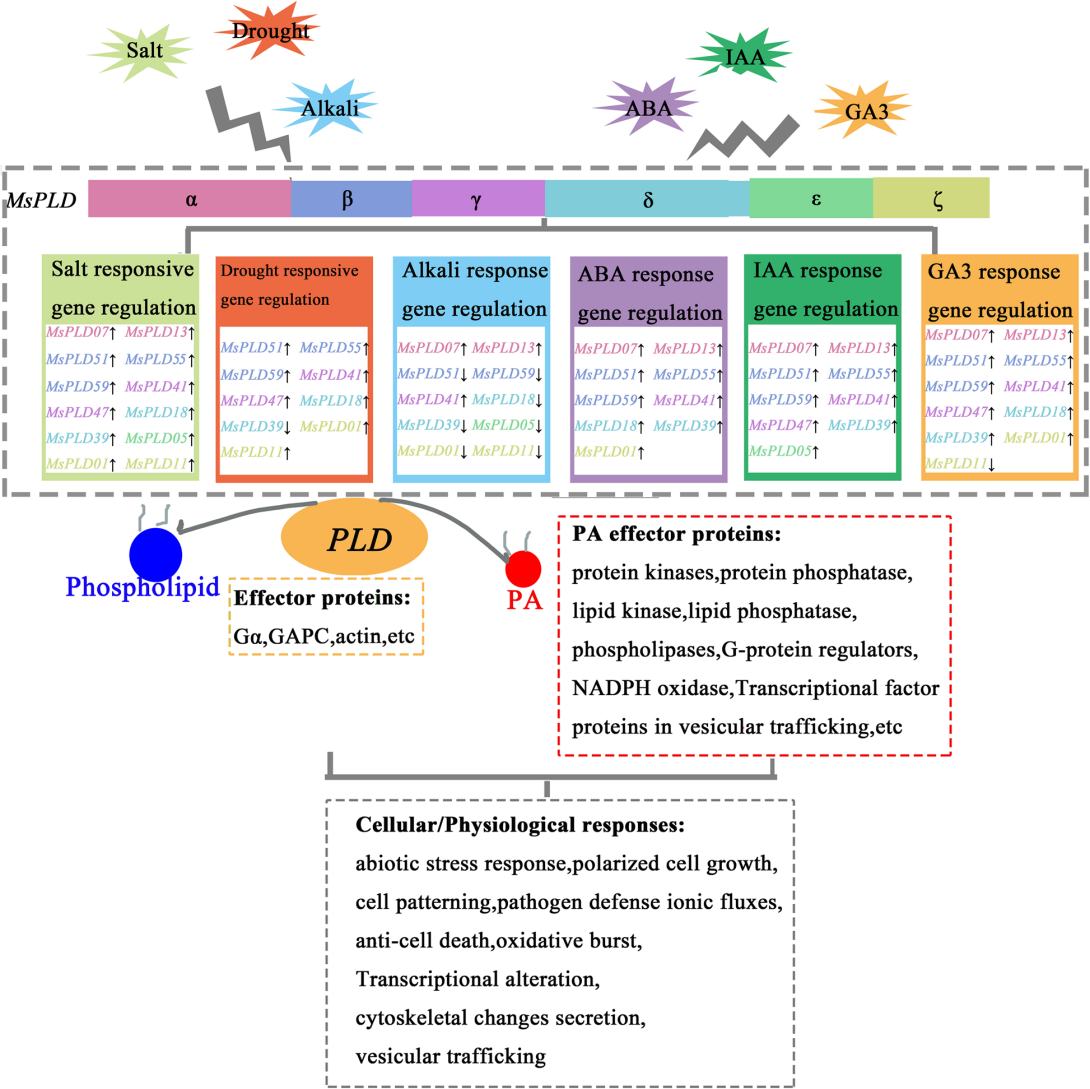
Model summarizing the roles of *MsPLDs* based on their gene expression profiles in alfalfa

In the present study, we found that *MsPLDs* show differences in terms of gene structure, predicted subcellular localization, and expression patterns. Moreover, *PLDs* can be activated in different ways, have distinguishable functions in Arabidopsis, and are involved in plant growth, development, and responses to multiple stresses. A model summarizing *MsPLDs* in response to abiotic stress and hormonal treatments is shown in Fig. 9, and this model is based on the results from previous studies [15, 46] as well as the present study. Different *PLDs* in alfalfa plants exhibit different expression patterns after abiotic stress and hormone treatments. *PLDs* can directly bind to effector proteins such as GAPC, Gα, and actin and indirectly interact with PA effector proteins such as protein kinases, protein phosphatase, and lipid kinase through PA, which can jointly participate in relevant physiological processes after stress and improve the plant’s ability to adapt to variable environments.

## Conclusion

In conclusion, a total of 59 *MsPLDs* were identified in the alfalfa genome, and these 59 *MsPLDs* were divided into 6 subtypes based on their phylogenetic relationships and 2 subfamilies based on their structural domains. Members of the same isoform have similar physicochemical properties, sequence structure, and domains. Duplications have likely been important for the expansion and evolution of *MsPLDs*. These *MsPLDs* contain *cis*-acting elements that respond to abiotic stress, hormones and light. A qRT-PCR analysis showed that most *MsPLDs* could respond positively to abiotic stress and hormone treatments, particularly to salt stress. To our knowledge, this study involves the first systematic and in-depth analysis of alfalfa *MsPLDs*, and these data provide a foundation for elucidating the molecular mechanism of *MsPLDs* underlying stress biology. This study expands the genetic resources for improving plant tolerance and serves as a reference for future functional investigations and molecular breeding in alfalfa.

## Materials and methods

### Identification and phylogenetic analysis of alfalfa *MsPLDs*

First, we obtained the hidden Markov model of the PF00164 domain from the Pfam 34.0 database (https://pfam.xfam.org/). The genome assembly files of alfalfa (cultivar XinJiangDaYe) [35] were downloaded from the website (https://figshare.com/projects/whole_genome_sequencing_and_assembly_of_Medicago_sativa/66380). We searched the alfalfa genome using HMMER 3.0 software [47] and used online sites such as Pfam [48] and InterPro (http://www.ebi.ac.uk/interpro/) for screening and identification. The protein sequences of the 12 *PLDs* of Arabidopsis were obtained from The Arabidopsis Information Resource (https://www.arabidopsis.org/index.jsp). A phylogenetic tree was generated with MEGA 6.0 using the maximum likelihood method with 1000 bootstrap replicates.

### Nucleotide and base sequence analysis

The chromosome location and sequence information of *MsPLDs* in alfalfa was obtained from the website (https://figshare.com/projects/whole_genome_sequencing_and_assembly_of_Medicago_sativa/66380). Protein sequences were analyzed using the ExPASy database (https://prosite.expasy.org/) to obtain information such as the relative molecular mass, amino acid length and theoretical isoelectric point. The protein sequences were analyzed for motifs and domains using MEME Suite 5.3.3 (https://meme-suite.org/meme/index.html) [49] and NCBI (https://www.ncbi.nlm.nih.gov/), respectively. All the analytical results were visualized using TBtools software [50]. In addition, amino acid sequence alignment was performed with DNAMAN. The *cis*-acting element of the promoter region 2000 bp upstream of the CDS region of *MsPLDs* was predicted using the PlantCARE (http://bioinformatics.psb.ugent.be) online analysis website [51].

### Collinearity and multiple synteny analysis of *MsPLDs*

The MCScanX tool was used to identify all tandem blocks and colinear gene pairs in the alfalfa genome, and relevant information about *MsPLDs* was extracted and visualized using TBtools [50]. In addition, the synonymous substitution rate (Ks) and nonsynonymous substitution rate (Ka) of *MsPLDs* and the Ks/Ka ratio were calculated with TBtools [50].

### Plant materials and treatments

In this study, alfalfa (cultivar XinJiangDaYe) was used as the plant material. Alfalfa seeds with uniform morphology and full seeds were selected, cultured in vermiculite and watered with 1/10 Hoagland nutrient solution. All plants were grown under a 16-h light/8-h dark photoperiod with day/night temperatures of 22 °C/18 °C. When the seeds were 4 weeks of age, plants with uniform growth were treated with 15% PEG-6000, 150 mmol/L NaCl, 150 mmol/L NaHCO_3_, 100 mmol/L ABA, 100 mmol/L IAA and 100 mmol/L GA3. Samples were collected at 0 h (CK) and 1 h, 3 h, 6 h, 12 h, and 24 h after treatment. Samples of roots, stems, mature leaves and young leaves were collected from one-month-old alfalfa for tissue-specific expression analysis. All sample collections were set up with three biological replicates. The samples were snap-frozen in liquid nitrogen after sampling, transferred to -80 °C and stored for subsequent analysis.

### Validation of *MsPLD* expression levels by qRT-PCR

Total RNA extraction and reverse transcription of the collected plant samples were performed based on the instructions provided with the kit. The quality of the reverse-transcribed cDNA was examined using the *GAPDH* gene as an internal reference. The reaction system was 1 µl of ddH_2_O, 5 µl of 2 × Phanta Max Master Mix, 1 µl of *GAPDH* S (10 µM), 1 µl of *GAPDH* AS (10 µM) and 1 µl of cDNA. The samples were loaded on the PCR system and subjected to the following conditions: 180 s at 95 °C followed by 40 amplification cycles consisting of denaturation for 10 s at 95 °C, annealing for 30 s at 58 °C, and extension for 30 s at 72 °C. The qRT-PCR experiments were performed using high-quality cDNA and 2 × Cham Q Universal SYBR q PCR Master Mix, and the rest of the reaction system and conditions were consistent with those used in the PCR analysis. Each reaction was performed with three replicates. The sequences of the internal reference primers are shown in Additional file 7. The relative levels of gene expression were determined by the 2^–ΔΔCT^ method [52].

### Yeast expression vector construction and the salt tolerance of *MsPLD05*

In this experiment, the coding sequence of the *MsPLD05* gene was obtained by PCR using the cDNA that was reverse transcribed from RNA extracted from alfalfa leaves under normal growth conditions. The *MsPLD05* fragment was ligated to pESC-HIS using T4 DNA ligase, and the recombinant expression plasmid was obtained and named pESC-HIS-MsPLD05. pESC-HIS and pESC-HIS-MsPLD05 were transformed into brewer’s yeast Y187 using the LiAc/SS-DNA/PEG transformation method [53]. The recombinant strains were screened on nutrient-deficient SD-HIS medium. Yeast strains transformed with pESC-HIS and pESC-HIS-MsPLD05 were isolated and then used for gradient dilution and inoculation into SD-HIS medium with NaCl (0, 0.5 and 1 mol/L). The results were obtained after 2 days of culture at 29 °C. The sequences of the primers are shown in Additional file 7.

### Statistical analysis

Statistical analysis was performed using SPSS statistics 22.0 software. Data were subjected to analysis of variance, and the means were compared using Student’s t-test at the 5% significance level.

## Supplementary Information

**Additional file 1. **Sequence list of 59 *MsPLDs*.

**Additional file 2.** Logos of 10 motifs in alfalfa.

**Additional file 3. **List of 10 motifs with basic information.

**Additional file 4. **Amino acid sequence alignment of 59 MsPLDs in alfalfa.

**Additional file 5. **List of synteny gene pairs and tandem duplication gene pairs of the MsPLD gene family.

**Additional file 6. **Sequences of primers used in qRT-PCR analysis.

**Additional file 7. **List of synonymous and nonsynonymous substitutions of MsPLD gene pairs in alfalfa.

## Data Availability

The data involved in this study are listed in the article and its additional files.

## Abbreviations

PLD: Phospholipase D
Ms: Medicago sativa
At: Arabidopsis thaliana
HMM: Hidden Markov Model
MEME: Multiple Em for Motif Elicitation
CDS: Coding domain sequence
qRT-PCR: Reverse transcription-quantitative PCR
Ka: Nonsynonymous substitution ratio
Ks: Synonymous substitution ratio
ABA: Abscisic acid
GA3: Gibberellic acid
IAA: Indol-1-yl-acetic acid

## References

[1] Wang G, Ryu S, Wang X (2012). Plant phospholipases: an overview. Methods Mol Biol.

[2] Wang X (2001). Plant phospholipases. Annu Rev Plant Physiol Plant Mol Biol.

[3] Fan L, Zheng S, Wang X (1997). Antisense suppression of phospholipase da retards abscisic acid- and ethylene-promoted senescence of postharvest Arabidopsis leaves. Plant Cell.

[4] Li M, Qin C, Welti R, Wang X (2006). Double knockouts of phospholipases Dzeta1 and Dzeta2 in Arabidopsis affect root elongation during phosphate-limited growth but do not affect root hair patterning. Plant Physiol.

[5] Li G, Lin F, Xue HW (2007). Genome-wide analysis of the phospholipase D family in Oryza sativa and functional characterization of PLD beta 1 in seed germination. Cell Res.

[6] Qin C, Wang X (2002). The Arabidopsis phospholipase d family. characterization of a calcium-independent and phosphatidylcholine-selective PLDζ1 with distinct regulatory domains. Plant Physiol.

[7] Zhao J, Zhou D, Zhang Q, Zhang W (2012). Genomic analysis of phospholipase D family and characterization of GmPLDαs in soybean (Glycine max). J Plant Res.

[8] Liu Q, Zhang C, Yang Y, Hu X (2010). Genome-wide and molecular evolution analyses of the phospholipase D gene family in poplar and grape. BMC Plant Biol.

[9] Du D, Cheng T, Pan H, Yang W, Wang J, Zhang Q (2013). Genome-wide identification, molecular evolution and expression analyses of the phospholipase D gene family in three Rosaceae species. Sci Hortic.

[10] Zheng L, Krishnamoorthi R, Zolkiewski M, Wang X (2000). Distinct Ca2+ binding properties of novel C2 domains of plant phospholipase dalpha and beta. J Biol Chem.

[11] Li M, Hong Y, Wang X (2009). Phospholipase D- and phosphatidic acid-mediated signaling in plants. Biochim Biophys Acta.

[12] Sung TC, Altshuller YM, Morris AJ, Frohman MA (1999). Molecular analysis of mammalian phospholipase D2. J Biol Chem.

[13] Sung TC, Zhang Y, Morris AJ, Frohman MA (1999). Structural analysis of human phospholipase D1. J Biol Chem.

[14] Pleskot R, Pejchar P, Bezvoda R, Lichtscheidl IK, Wolters-Arts M, Marc J, Zarsky V, Potocky M (2012). Turnover of phosphatidic acid through distinct signaling pathways affects multiple aspects of pollen tube growth in tobacco. Front Plant Sci.

[15] Wang X, Su Y, Liu Y, Kim S-C, Fanella B. Phosphatidic acid as lipid messenger and growth regulators in plants. Phospholipases Plant Signal. 2014;20:69–92.

[16] Deinlein U, Stephan AB, Horie T, Luo W, Xu G, Schroeder JI (2014). Plant salt-tolerance mechanisms. Trends Plant Sci.

[17] Julkowska MM, Testerink C (2015). Tuning plant signaling and growth to survive salt. Trends Plant Sci.

[18] Welti R, Li W, Li M, Sang Y, Biesiada H, Zhou HE, Rajashekar CB, Williams TD, Wang X (2002). Profiling membrane lipids in plant stress responses. Role of phospholipase D alpha in freezing-induced lipid changes in Arabidopsis. J Biol Chem.

[19] Li W, Li M, Zhang W, Welti R, Wang X (2004). The plasma membrane-bound phospholipase Ddelta enhances freezing tolerance in Arabidopsis thaliana. Nat Biotechnol.

[20] Bargmann BO, Laxalt AM, ter Riet B, van Schooten B, Merquiol E, Testerink C, Haring MA, Bartels D, Munnik T (2009). Multiple PLDs required for high salinity and water deficit tolerance in plants. Plant Cell Physiol.

[21] Guo L, Devaiah SP, Narasimhan R, Pan X, Zhang Y, Zhang W, Wang X (2012). Cytosolic glyceraldehyde-3-phosphate dehydrogenases interact with phospholipase Ddelta to transduce hydrogen peroxide signals in the Arabidopsis response to stress. Plant Cell.

[22] Lu S, Bahn SC, Qu G, Qin H, Hong Y, Xu Q, Zhou Y, Hong Y, Wang X (2013). Increased expression of phospholipase Dalpha1 in guard cells decreases water loss with improved seed production under drought in Brassica napus. Plant Biotechnol J.

[23] Hong Y, Devaiah SP, Bahn SC, Thamasandra BN, Li M, Welti R, Wang X (2009). Phospholipase D epsilon and phosphatidic acid enhance Arabidopsis nitrogen signaling and growth. Plant J.

[24] Hong Y, Lu S (2014). Phospholipases in plant response to nitrogen and phosphorus availability.

[25] Zhao J, Devaiah SP, Wang C, Li M, Welti R, Wang X (2013). Arabidopsis phospholipase Dbeta1 modulates defense responses to bacterial and fungal pathogens. New Phytol.

[26] Hong Y, Zhao J, Guo L, Kim SC, Deng X, Wang G, Zhang G, Li M, Wang X (2016). Plant phospholipases D and C and their diverse functions in stress responses. Prog Lipid Res.

[27] Zhao J, Wang X (2004). Arabidopsis phospholipase Dalpha1 interacts with the heterotrimeric G-protein alpha-subunit through a motif analogous to the DRY motif in G-protein-coupled receptors. J Biol Chem.

[28] Simoes I, Mueller EC, Otto A, Bur D, Cheung AY, Faro C, Pires E (2005). Molecular analysis of the interaction between cardosin A and phospholipase D(alpha). Identification of RGD/KGE sequences as binding motifs for C2 domains. FEBS J.

[29] Zhang Y, Zhu H, Zhang Q, Li M, Yan M, Wang R, Wang L, Welti R, Zhang W, Wang X (2009). Phospholipase dalpha1 and phosphatidic acid regulate NADPH oxidase activity and production of reactive oxygen species in ABA-mediated stomatal closure in Arabidopsis. Plant Cell.

[30] Anthony RG, Henriques R, Helfer A (2004). sza´ros TsM, Rios G, Testerink C, Munnik T, k MD, Koncz C, gre LsB: a protein kinase target of a PDK1 signalling pathway is involved in root hair growth in Arabidopsis. EMBO J.

[31] Yu L, Nie J, Cao C, Jin Y, Yan M, Wang F, Liu J, Xiao Y, Liang Y, Zhang W (2010). Phosphatidic acid mediates salt stress response by regulation of MPK6 in Arabidopsis thaliana. New Phytol.

[32] Guo L, Mishra G, Markham JE, Li M, Tawfall A, Welti R, Wang X (2012). Connections between sphingosine kinase and phospholipase D in the abscisic acid signaling pathway in Arabidopsis. J Biol Chem.

[33] Radovic J, Sokolovic D, Markovic J (2009). Alfalfa-most important perennial forage legume in animal husbandry. Biotechnol Anim Husb.

[34] Li X, Brummer EC (2012). Applied genetics and genomics in alfalfa breeding. Agronomy.

[35] Chen H, Zeng Y, Yang Y, Huang L, Tang B, Zhang H, Hao F, Liu W, Li Y, Liu Y (2020). Allele-aware chromosome-level genome assembly and efficient transgene-free genome editing for the autotetraploid cultivated alfalfa. Nat Commun.

[36] Wei Y, Shi H, Xia Z, Weiwei T, Ding Z, Yan Y, Wang W, Hu W, Li K (2016). Genome-wide identification and expression analysis of the WRKY gene family in cassava. Front Plant.

[37] Hong Y, Pan X, Welti R, Wang X (2008). Phospholipase Dalpha3 is involved in the hyperosmotic response in Arabidopsis. Plant Cell.

[38] Mane SP, Vasquez-Robinet C, Sioson AA, Heath LS, Grene R (2007). Early PLDalpha-mediated events in response to progressive drought stress in Arabidopsis: a transcriptome analysis. J Exp Bot.

[39] Eliáš M, Potocký M, Cvrčková F, Žárský V (2002). Molecular diversity of phospholipase D in angiosperms. BMC Genomics.

[40] Abdullah, Faraji S, Mehmood F, Malik HMT, Ahmed I, Heidari P, Poczai P (2021). The GASA gene family in Cacao (Theobroma cacao, Malvaceae): genome wide identification and expression analysis. Agronomy.

[41] Musavizadeh Z, Najafi-Zarrini H, Kazemitabar SK, Hashemi SH, Faraji S, Barcaccia G, Heidari P (2021). Genome-wide analysis of potassium channel genes in rice: expression of the *OsAKT* and *OsKAT* genes under salt stress. Genes (Basel).

[42] Noji M (1998). Isoform-dependent differences in feedback regulation and subcellular localization of serine acetyltransferase involved in cysteine biosynthesis from Arabidopsis thaliana. J Biol Chem.

[43] Katagiri T, Takahashi S, Shinozaki K (2001). Involvement of a novel Arabidopsis phospholipase D, AtPLDα, in dehydration-inducible accumulation of phosphatidic acid in stress signalling. Plant J.

[44] Zhang Q, Lin F, Mao T, Nie J, Yan M, Yuan M, Zhang W (2012). Phosphatidic acid regulates microtubule organization by interacting with MAP65-1 in response to salt stress in Arabidopsis. Plant Cell.

[45] Hong Y, Zhang W, Wang X (2010). Phospholipase D and phosphatidic acid signalling in plant response to drought and salinity. Plant Cell Environ.

[46] Wang X (2005). Regulatory functions of phospholipase D and phosphatidic acid in plant growth, development, and stress responses. Plant Physiol.

[47] Potter SC, Aurélien L, Eddy SR, Youngmi P, Rodrigo L, Finn RD (2018). HMMER web server: 2018 update. Nucleic Acids Res.

[48] Sigrist CJA, Cerutti L, Hulo N, Gattiker A, Falquet L (2002). PROSITE: A documented database using patterns and profiles as motif descriptors. Brief Bioinform.

[49] Enis A, Dannon B, Marius V, Daniel B, Dave B, Martin Č, John C, Dave C, Nate C, Carl E (2016). The galaxy platform for accessible, reproducible and collaborative biomedical analyses: 2016 update. Nucleic Acids Res.

[50] Chen C, Chen H, Zhang Y, et al. TBtools: an integrative toolkit developed for interactive analyses of big biological data. Mol Plant. 2020;13(8):1194–202. 10.1016/j.molp.2020.06.009 32585190

[51] Rombauts S, Dehais P, Van Montagu M, Rouze P (1999). PlantCARE, a plant cis-acting regulatory element database. Nucleic Acid Res.

[52] Kenneth JL, Thomas DS (2002). Analysis of relative gene expression data using real-time quantitative PCR and the 2-ΔΔCT method. Methods.

[53] Gietz RD, Schiestl RH, Willems AR, Woods RA (2010). Studies on the transformation of intact yeast cells by the LiAc/SS-DNA/PEG procedure. Yeast.

