# Supplementary figures and images for: Genome-wide investigation of the PLD gene family in alfalfa (Medicago sativa L.): identification, analysis and expression
Source: BMC Genomics. 2022 Mar 28;23:243. doi: 10.1186/s12864-022-08424-9 (PMC8962232; doi:10.1186/s12864-022-08424-9)

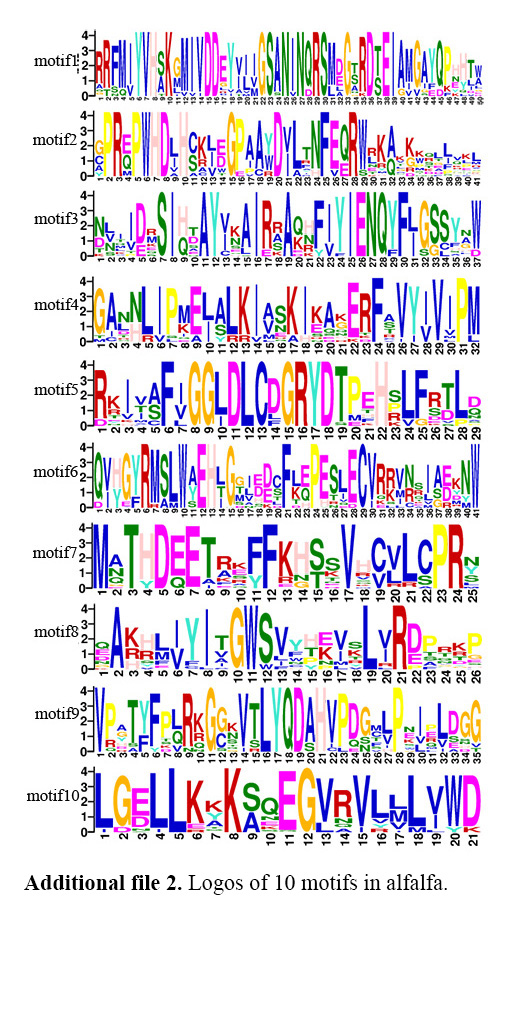

Supplement: Supplementary file 2 — Additional file 2. Logos of 10 motifs in alfalfa. [file 12864_2022_8424_MOESM2_ESM.jpg]

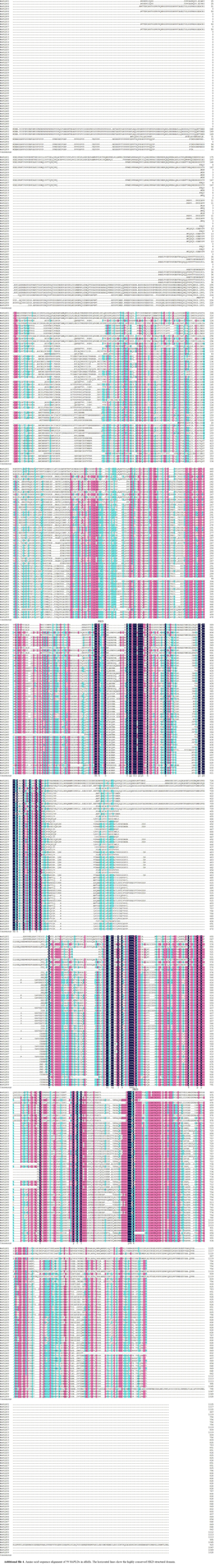

Supplement: Supplementary file 4 — Additional file 4. Amino acid sequence alignment of 59 MsPLDs in alfalfa. [file 12864_2022_8424_MOESM4_ESM.jpg]
